# Cardiorenal Effects of Newer NSAIDs (Celecoxib) versus Classic NSAIDs (Ibuprofen) in Patients with Arthritis

**DOI:** 10.1155/2011/862153

**Published:** 2011-05-31

**Authors:** Ragia Hegazy, Mohamed Alashhab, Madiha Amin

**Affiliations:** ^1^Department of Forensic Medicine and Clinical Toxicology, Benha Faculty of Medicine, Benha University, Benha, Egypt; ^2^Department of Pharamacology and Toxicology, Faculty of Pharmacy, Umm Al-Qura University (UQU), Mecca, Saudi Arabia; ^3^Department of Orthopedics, Benha Faculty of Medicine, Benha University, Benha, Egypt; ^4^Department of Pharmacognocy, Alexandria Faculty of Pharmacy, Alexandria University, Alexandria, Egypt; ^5^Department of Pharmacognocy, Faculty of Pharmacy, Umm Al-Qura University (UQU), Mecca, Saudi Arabia

## Abstract

*Background*. Arthritis is a common condition that co-exists in the elderly population. This condition leads to frequent administration of comorbid analgesics especially non steroidal anti-inflammatory drugs (NSAIDs). *Aim*. To study cardiorenal toxicity of celecoxib versus ibuprofen in arthritic patients. *Subjects and Methods*. Seven hundred ninety-wo arthritic patients were enrolled in the study for 6 months. Three hundred ninety-six patients administered celecoxib 400 mg twice a day; 396 patients administered ibuprofen 300 mg three times a day. Effects measured included investigator-reported hypertension, edema, or congestive heart failure, increases in serum creatinine or reduction in serum creatinine clearance, and changes in serum electrolytes. *Results*. Celecoxib was associated with significant (*P* < .05) lower incidence of hypertension and edema in comparison with ibuprofen. Systolic hypertension occurred significantly less (*P* < .05) with celecoxib compared with ibuprofen. Serum creatinine was significantly increased (*P* < .05) in patients treated with ibuprofen in comparison with celecoxib. Creatinine clearance was significantly lower (*P* < .05) in cases treated with ibuprofen in comparison to celecoxib. Nonsignificant changes in serum body electrolytes occurred. *Conclusion*. The most important finding of this study was the lowering incidence of cardiorenal toxicity of celecoxib in comparison with ibuprofen.

## 1. Introduction

Nonsteroidal anti-inflammatory drugs, NSAIDs are widely used drugs valued for their anti-inflammatory, analgesic and antipyretic properties. However, Classic (nonselective,) NSAIDs work by inhibiting two isoforms of the enzyme Cyclo-oxygenase (COX): COX-1 and COX-2. COX-1 is expressed continuously in most body tissues and in the stomach catalyses the production of prostaglandins which safeguard the gastric mucosa. Inhibition of COX-1 is responsible for NSAID-induced gastrointestinal damage [1]. COX-2 is much less widely expressed but is induced by proinflammatory stimuli and catalyses prostaglandins mediating the inflammatory response. Anti-inflammatory action is derived from inhibition of COX-2 [2]. 

It is believed that selective COX-2 inhibitors (COXIBs) have reduced side effects especially gastrointestinal and platelet-related adverse events in comparison to classic NSAIDs. Celecoxib was the first COX-2 selective inhibitors to be developed. At full therapeutic doses, celecoxib possesses comparable analgesic and inflammatory efficacy to nonselective NSAIDs in the treatment of rheumatoid arthritis (RA) and osteoarthritis (OA) [3–5].

Non-selective inhibition of prostaglandins synthesis is associated with antinatriuretic and vasoconstrictor effects and frequently reduces glomerular filtration rate (GFR). The sensing mechanisms of sodium are largely through the COX-2 enzymatic pathway located in the macula densa of the kidney. The inhibition of this enzyme will lead to antinatriuretic effect that is manifested clinically by edema, and frequently linked with, destabilization of blood pressure (BP) control in treated hypertensive individuals. In addition, it may subsequently induce congestive heart failure in susceptible individual, so the use of nonselective NSAIDs may result in acute hemodynamically mediated deterioration of renal functions in one of five patients within several days of NSAIDs use. This is fully reversible following discontinuation of the offending drug. Other syndromes are extremely rare; they include other electrolyte complications such as hyperkalemia, and hyponatremia, nephritic syndrome with interstitial nephritis [6, 7]. 

On the other hand, the use of selective COX-2 in healthy individuals is associated with mild oedema and hypertension due to modest sodium retention in the first one to two days of therapy. It is followed by a return to baseline excretion levels and elimination of retained salt and water after another one to two days without drug discontinuation. It may be hypothesized that a selective COX-2 inhibitors (celecoxib) may have decreased renal adverse effects relative to non-selective inhibitors [8, 9]. 

The celecoxib long-term arthritis safety study (CLASS) was undertaken to compare the long term upper gastrointestinal tract of celecoxib 400 mg twice daily (b.i.d.) two to four times the full therapeutic doses for RA and OA, respectively, with normal full therapeutic doses of one of classic NSAIDs, for example, ibuprofen 800 mg three times a day (t.d.s.). The CLASS trial proved that celecoxib is safer to gastrointestinal tract in comparison to non-selective COX inhibitors [10].

 Cardiorenal safety of celecoxib compared to ibuprofen according to the doses used in the CLASS trial is used in this study. 

### 1.1. Aim of the Work

The purpose of this study was to examine the Cardiorenal effects of a representative agent from different classes of NSAIDs; celecoxib represent the selective COX inhibitors, and ibuprofen represents the non-selective COX inhibitors.

## 2. Subjects and Methods

### 2.1. Subjects

This study was conducted on 792 patients from the orthopedic outpatient clinics Al-Salam hospital, Jeddah, Saudia Arabia.

### 2.2. Inclusion Criteria 

Patients with a history of RA or OA with average of 40–70 years, were divided into 2 groups.

**Group I**

This group included 396 patient's administered celecoxib (selective COX2 inhibitor) at supratherapeutic dose, that is, 400 mg b.i.d. The supratherapeutic dose equals two- to four-fold greater than the maximum effective doses for RA and OA.

**Group II**

This group included 396 patients administered ibuprofen (non-selective COX inhibitor) at therapeutic dose, that is, 800 mg t.d.s.

The used doses were commonly prescribed and indicated for use by FDA and CLASS trial.

An institutional review board (IRB) approval was obtained for the study. Informed consent was given to all participants before enrollment to read and being discussed with the investigator and staff. All participants signs on informed consent on entering the study.

 After baseline visit and administration of the initial dose of study medication, followup took place every two weeks for two months, and then every 4 weeks thereafter, until study termination. All patients took study medication for 6 months from October 1st 2006 to April 1st 2007.

### 2.3. Exclusion Criteria

- underlying peptic ulcer
- history of myocardial infarction
- stroke
- cerebrovascular accident
- transient ischemic attack (TIA)
- renal insufficiency in 6 months before the study, that is, serum creatinine above 1.5 mg/dL
- any medications that could directly or indirectly affect renal functions.

### 2.4. Methods

In each visit, each patient in the two groups was subjected to the follwing:

1. questionnaires to collect relevant data for, example, any experienced symptoms that were not associated with patients' arthritis since the last visit
2. careful physical examination with special concern to the following:
  - body weight at least ≥3% increase was considered positive finding,
  - vital signs especially blood pressure with record of the positive cases, that is, increase in systolic >20 mm/Hg, increase in base line or absolute level above 140 mm/Hg, and for diastolic blood pressure >15 mm/Hg, increase in baseline or absolute level above 90 mm/Hg,
  - edema whether peripheral or generalized by inspection and palpation through pressing the skin over the lateral melleolus of the tibia in the leg,
  - manifestations of congestive heart failure (CHF): generalized edema, congested neck veins, and enlarged tender liver.
3. laboratory investigations.

Blood samples: in each visit, 25 *μ*L of blood were drawn up from each patient and used to measure the levels of the following.

#### 2.4.1. Blood Urea Nitrogen (BUN)

 Each case had the BUN ≥20 mg/dL defined as mild prerenal azotemia and completes the other kidney function tests.

Blood urea nitrogen (BUN) was assayed by colorimetric method described by Patton and Crouch [11] using a commercial kit of Bio-Merieux (France). Normal value: 18–33 mg/dL.

#### 2.4.2. Serum Creatinine Level

The aim was to detect any increase in creatinine level by 0.5 mg/dL over baseline or any value above 1.5 mg/dL during the study.

 Serum creatinine was assayed by colorimetric method described by Henry [12] using a commercial kit of Diamond Diagnostics (Egypt). Normal value: 0.8–2.0 mg/dL.

#### 2.4.3. Creatinine Clearance

(as calculated by Cockcroft Gaunt formula) = 140 − (age × weight)÷(serum  creatinine × 72).

 If ≥30% from the baseline during treatment, it should be analyzed as well [12].

#### 2.4.4. Serum Electrolytes

They included sodium, potassium, chloride, and bicarbonate [12].

## 3. Statistical Method [13]

The methods used for statistical analysis of the collected data were the following:

1. *t*-test for comparison between the means of two means ± standard deviations, and *P* values less than  .05 was considered significant,
2. one-way ANOVA test (*F* test) for comparison between more than two means ± standard deviations.

## 4. Results

**Figure 1:**
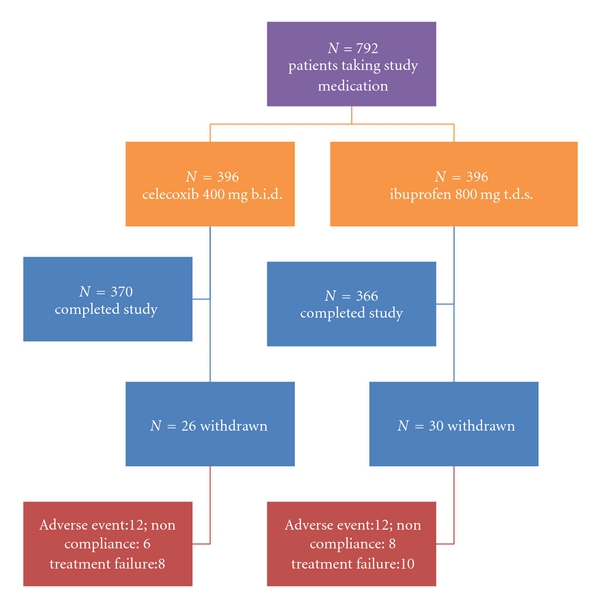
patient disposition in the study. Of 792 patients randomized and took study medication (ITT cohort), 736 patients completed the study. The most common reason for withdrawal from the trial was an adverse event, followed by lack of drug efficacy and non-compliance with the protocol.

As Figure 1 shows, a total number of 792 patients were randomized, of whom received at least one dose of the study drug, and were, therefore, included in the intent-to-treat (ITT) population (RA: *N = *290; OA: *N = *502). In the ITT population, 396 patients received celecoxib, and 396 were treated with ibuprofen.

Mean patient's age was about 55 years in each treatment group; most (69%–71%) patients were female, and mean BP was 133/80 mm/Hg. As determined by medical history, slightly less than 40% of patients of each treatment group had preexisting hypertension. Overall BP levels and renal function were normal at baseline and comparable between treatment groups; see (Table 1).

**Blood Presuure (Table 2)**

Celecoxib was associated with an incidence of hypertension (both new-onset and aggravated) that was significantly lower than standard doses of ibuprofen (*P* < .05). Withdrawals from study treatment owing to hypertension-related adverse events were uncommon and occurred with similar frequency among the two treatment groups. In patients taking ibuprofen, the increased incidence of hypertension versus celecoxib was accompanied by a corresponding trend towards a mean increase in systolic BP (*P* = .09). Ibuprofen treatment was associated with a significantly (*P* < .05) greater proportion of patients with systolic elevation >20 mm/Hg and above 140 mm/Hg as compared with celecoxib. No treatment differences were observed for diastolic BP.

**Fluid Retention (Table 3)**

**Figure 2:**
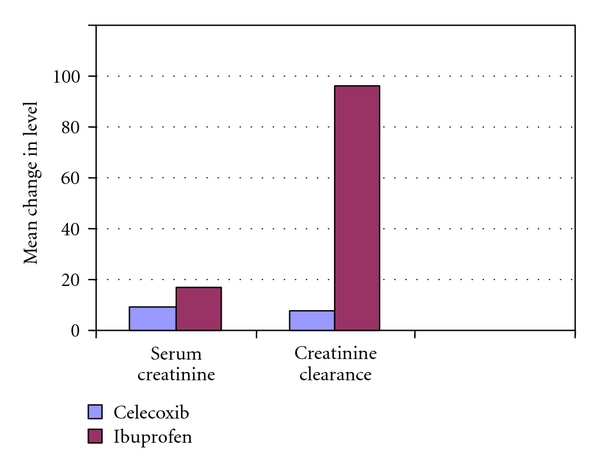
Renal effects of celecoxib versus ibuprofen.

Ceceloxib was associated with significantly lower incidence of investigator-reported edema (including either peripheral or generalized edema) compared with ibuprofen (*P* < .05) (see Figure 2).

Adverse events of CHF were uncommon as reported by investigators (approximately one-tenth the rate of overall edema, and no significant differences were detected between treatments). Withdrawals from study treatment owing to either edema or CHF were uncommon overall, and no significant treatment-related differences were detected. In addition, no significant treatment differences were observed with respect to body weight gain ≥3%.

**Renal Functions**

Renal functions of enrolled patients were normal, as reflected by baseline serum creatinine levels or estimated creatinine clearance; see (Table 1). Nearly all patients (>99%) had a serum creatinine ≤1.5 mg/dL, which reflect normal function required for protocol.

Renal functions, as reflected by serum creatinine levels and estimated creatinine clearance, decreased significantly in patients assigned to ibuprofen compared with those receiving celecoxib (*P* < .05) (Table 4).

Clinically important renal dysfunction, defined as (1) increases in serum creatinine > 0.5 mg/dL relative to baseline assessment or serum creatinine >1.5 mg/dL at any post-baseline assessment or (2) a decrease in estimated creatinine clearance ≥30% relative to baseline are provided in.

For the entire treatment cohort, one significant treatment difference was detected: the incidence of ≥30% reductions in estimated creatinine clearance from baseline was significantly lower in treatment with celecoxib as compared with ibuprofen (Table 5).

An evaluation of the time course of these changes revealed that the highest frequency occurred early after initiation of treatment (week 4 and 8 clinic visits) and declined thereafter, rather than progressively increasing risk as a function of duration of treatment of the two treatment groups. It should be noted, however, that a substantial proportion of patients exhibited these changes 3 months or longer after treatment was initiated, suggesting that the hazard, although perhaps diminishing with time, does continue through the therapy period. Increases in serum creatinine of >0.1 mg/dL were uncommon, occurring in seven celecoxib-treated patients and nine patients taking ibuprofen. These differences were not significantly different. For those patients characterized by mild prerenal azotemia at baseline (serum blood urea nitrogen >20 mg/dL) but relatively normal renal functions; see (Table 5), the incidence of clinically important changes in renal function was at least two-fold lower (*P* < .05) in patients receiving ceclecoxib compared with ibuprofen-treated patients.

Treatment differences in mean serum creatinine and estimated creatinine clearance were also observed in this cohort; see (Table 5). No differences were observed between treatment groups for patients without baseline azotemia (i.e., with BUN ≤20 mg/dL).

**Figure 3:**
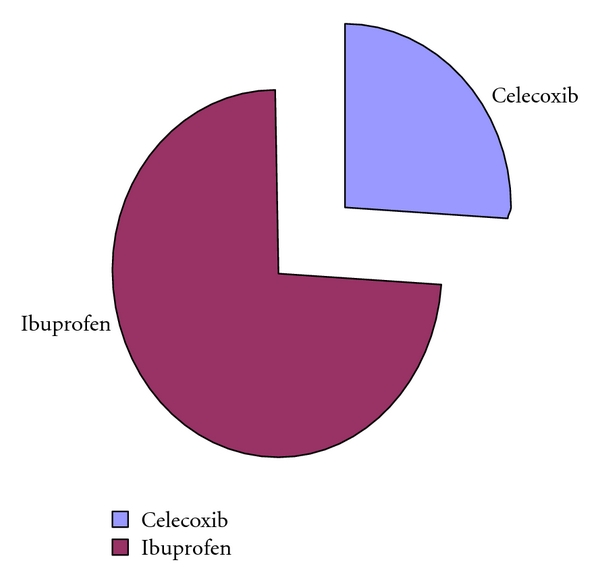
Whithdrawal form the study due to adverse event.

Patients with normal serum creatinine/prerenal azotemia at baseline were significantly more likely to withdraw from the study owing to an adverse event if their creatinine level increased to >1.5 mg/dL on treatment versus the overall cohort (relative risk, 2.0; *P* < .05). When expressed as percentage of the patients with normal serum creatinine/prerenal azotemia at baseline, the withdrawal rate in patients treated with celecoxib (1.5%) was significantly lower compared with those receiving ibuprofen (4.2%; *P* < .05) as shown in Figure 3.

**Electrolyte and Acid Base**

Changes in serum levels of sodium, potassium, chloride, and bicarbonate were minimal between treatment groups and were not of clinical significance.

**Serious Cardiorenal-Related Adverse Events**

The incidence of serious cardiorenal-related adverse events during treatment with celecoxib or ibuprofen was low. The study investigator reported one case of uremia in an ibuprofen-treated patient. Two cases of hyponatremia were reported in patients treated with celecoxib, and one case was reported in a patient taking ibuprofen. No cases of serious adverse events related to nephrotic syndrome, interstitial nephritis, or papillary necrosis were observed.

## 5. Discussion

The large cardiorenal database from the CLASS trial provided the opportunity to observe and compare the natural history of the onset and clinical management of cardiorenal related adverse events that evolve when exposing a typical adult arthritic population to chronic treatment with non-selective NSAIDs (ibuprofen) or supratherapeutic doses of COXIB (celecoxib) [14]. Thus, this study provides a new and important clinical resource of therapeutic cardiorenal safety information. The data have permitted the description of a clinical paradigm, where in certain key patient characteristics, it may provide additional predictive clinical information with respect to future drug-specific susceptibility to impairment of cardiorenal function.

The present analysis support the hypothesis that rheumatoid arthritis (RA) and osteoarthritis (OA) patients receiving celecoxib 400 mg b.i.d. have an overall reduced risk of cardiorenal-related adverse events compared with standard therapeutic doses of the nonselective NSAIDs, ibuprofen 800 mg t.d.s. In particular, celecoxib treatment was superior to the nonselective NSAIDs with respect to renal function in patients with prerenal compromise. Some important differences were observed in the cardiorenal safety profiles of celecoxib versus nonselective NSAIDs. Compared with celecoxib, the incidence of adverse events related to hypertension and edema was significantly higher with ibuprofen, and the decline in renal functions was also more apparent with ibuprofen. These data provide a greater understanding of the relative effects of these agents on cardiorenal function and homeostasis. 

The increased incidence of hypertension in patients taking ibuprofen versus celecoxib appeared to be largely a function of effects on systolic, rather than diastolic, BP. Ibuprofen was associated with significantly higher incidences of clinically meaningful elevations in systolic BP versus celecoxib. The importance of systolic BP has been highlighted in ALLAHT [15], Kostis et al. [16], and Steassen et al. [17]. These studies showed that elevated systolic BP is associated with heart failure, stroke, myocardial infarction, and death. 

Whelton et al. [18] evidenced now that destabilization of BP in treated hypertensive patients, by rofecoxib, is a significant risk marker for the development of acute myocardial infarction and stroke events. Much additional information with respect to the latter issues and the emergence that some NSAIDs may also cause cardiotoxicity is needed [19].

 In the present study, longterm treatment with supratherapeutic doses of celecoxib in the CLASS trial had a little effect on BP levels in these arthritis patients. This finding is supported by previous studies demonstrating that celecoxib does not affect mean systolic BP changes in hypertensive patients [20, 21].

In contrast, many NSAIDs and COXIBs, such as rofecoxib, antagonize the BP-lowering effects of certain antihypertensive agents, in particular, angiotensin-converting enzyme inhibitors and *β*-blockers [22].

In the present analysis, celecoxib was superior to the ibuprofen studied with respect to renal dysfunction. Ibuprofen appeared to cause a significantly higher rate of decline in renal functions than celecoxib. This may suggest a disproportionate effect of ibuprofen on renal perfusion. Importantly, in patients with prerenal azotemia (but normal serum creatinine) at baseline, celecoxib was associated with a lower-rate deterioration of renal functions compared with ibuprofen. However, as some renal dysfunction was evident in the celecoxib treatment group, caution should still be used when treating patients at risk of adverse renal events. Changes in electrolytes levels were small and were not of clinical relevance.

In healthy human subjects on normal diets, COX-2 inhibitors have minimal effects on renal hemodynamics [23, 24] that COX-2 inhibitors decrease GFR in salt-depleted subjects [25–27]. Since nitric oxide (NO) regulates renal cortical COX-2 expression [28], Lopez et al. [29] proposed that in addition to the salt depletion and prolonged duration of COX-2 inhibition, reduced NO may be another factor underlying these effects of COX-2-specific inhibitors on renal hemodynamic.

It is evident from this study that there are differential cadiorenal effects associated with celecoxib versus nonselective NSAIDs, ibuprofen. Previous studies have also shown that cadiorenal safety profiles are not consistent across the class of COXIBs. For example, rofecoxib treatment is associated with dose-dependent increases in hypertension and edema [30–32]. 

 In two recent, randomized double-blind trials for 6 weeks of OA patients taking antihypertensive agents, the study participants taking celecoxib 200 mg daily were less likely to experience renal adverse events than the group receiving rofecoxib 25 mg daily [33].

The differing renal safety profiles across the classes of nonselective NSAIDs and COXIBs suggest that some of the renal effects of the latter may be “molecule-specific” rather than as a result of a “class effect” of COX-2 inhibition [34].

This study suggested that the clinical management of arthritis patients may be impacted by the agent selected to treat their pain and by the patient's underlying health status with regard to cadiorenal side effects. Our results indicate that cadiorenal side effects are relatively common with NSAID treatment and require careful medical monitoring of patients together with other safety-related parameters (e.g., hepatotoxicity associated with some NSAIDs such as diclofenac).

Anti-inflammatory drug-induced effects on BP or renal function should be cautiously monitored clinically during the use of such drugs. These adverse renal events may be selectively reduced or minimized by the selection of an anti-inflammatory drug, such as celecoxib, that appears to have the least detrimental effects upon renal function, such as demonstrated in this study.

The present analysis had some potential limitations. Many OA and RA patients were elderly and had significant comorbidity, including cardiovascular disease, hypertension, and abnormal renal functions. However, our study did not include patients with abnormal renal functions at baseline, and results could not apply to these patients. Another limitation in the present analysis was that patients in this study were not stratified by baseline body weight, which may impact significantly on baseline creatinine levels.

## 6. Conclusion

Supratherapeutic dose of celecoxib was associated with a cardiorenal safety profile compared with standard doses of ibuprofen.

## 7. Recommendations

1. Further work is needed to study the renal safety profile of nonspecific NSAIDs and COXIBs in patients with abnormal renal function.
2. Physicians need to be alerted to effects of NSAIDs on BP and kidney functions.
3. Celecoxib should be given to arthritic patients with prerenal azotemia and classic NSAIDs should be avoided.

## Figures and Tables

**Table 1:** Baseline characteristics and exposure to treatment.

	Celecoxib 400 mg b.i.d. (*N = *396)	Ibuprofen 800 mg t.d.s. (*N = *396)
Exposure to treatment median (days)	182	176

Age		
Mean (years)	55.2	54.9
60–70 years (%)	26.6	25.5
≥70 years (%)	5.2	3.9

Female	68.5	70.8

History (%)		
Hypertension	39.0	38.1
Diabetes	8.8	8.1

Creatinine		
Serum level (mg/dL)	0.79	0.77
Clearance (mL/min.)*	112.2	116.4

b.i.d. twice a day; t.d.s. three times a day.

*estimated by Cockroft-Gault formula.

**Table 2:** Blood pressure effects of celecoxib versus ibuprofen.

Event	Celecoxib 400 mg b.i.d. (*N = *396)	Ibuprofen 800 mg t.d.s. (*N = *396)
Any hypertension adverse event (% of patients)^§^	2.7	4.2*
New-onset hypertension (% of patients)	2.0	3.1*
Aggravated hypertension (% of patients)	0.8	1.2

Withdrawal for hypertension related adverse effects (% of patients)^§^	0.3	0.3

Mean change in blood pressure (mmHg)		
Systolic	−0.6	>20*
Diastolic	−0.7	3.2

Increases in blood pressure (% of patients)^#^		
Systolic >20 mmHg from baseline and absolute value > 140 mmHg	5.0	7.0*
Diastolic >15 mmHg from baseline and absolute value > 90 mmHg	1.9	2.2

b.i.d. twice a day; t.d.s. three times a day.

^§^New-onset or aggravated.

^#^observed at final clinic visit.

*P <.05 versus celecoxib by Fisher exact test.

**Table 3:** Fluid retention effects of celecoxib versus ibuprofen.

	Celecoxib 400 mg b.i.d. (*N = *396)	Ibuprofen 800 mg t.d.s. (*N = *396)
Any edema-related adverse events (% of patients)^§^	4.1	6.2*
CHF Congestive Heart Failure (% of patients)	0.5	0.5
Withdrawals for edema-related adverse events (% of patients)	0.7	1.0
Withdrawals due to CHF (% of patients)	0.1	0.3
Increase in body weight of ≥3% (% of patients)	20.7	21.1

b.i.d. twice a day; t.d.s. three times a day.

^§^Includes investigator reports of edema, generalized edema or peripheral edema.

*P < .05 versus celecoxib by Fisher exact test.

**Table 4:** Renal effects of celecoxib versus ibuprofen.

	Celecoxib 400 mg b.i.d. (*N = *396)	Ibuprofen 800 mg t.d.s. (*N = *396)
Mean change in serum creatinine (mg/dL)	0.009 ± 0.002	0.027 ± 0.004*
Mean change in estimated creatinine clearance (ml/min)	0.08 ± 0.37	−2.82 ± 0.51*

b.i.d. twice a day; t.d.s. three times a day.

*P < .05 versus celecoxib by Fisher exact test.

**Table 5:** Renal functions in patients with prerenal azotemia at baseline (BUN > 20 mg/dL).

Comparison item	Celecoxib 400 mg b.i.d. (*N = *126) Of total 396	Ibuprofen 800 mg t.d.s (*N = *146) Of total 396
Baseline serum creatinine (mg/dL)	0.93	0.94
Estimated baseline creatinine clearance (mg/dL)	91.1	91.3
Mean change in serum creatinine (mg/dL)	0.003 ± 0.007	0.049 ± 0.012*
Mean change in estimated creatinine clearance (ml/min.)	1.27 ± 0.69	−1.65 ± 1.03*
Withdrawal of study owing to adverse event	1.5	4.2*

b.i.d. twice a day; t.d.s. three times a day; BUN: blood urea nitrogen.

*P < .05 by analysis covariance by Fisher exact test.
